# Rapid Smile Restoration: Basal Implants for the Edentulous Mandible With Immediate Loading

**DOI:** 10.7759/cureus.62655

**Published:** 2024-06-18

**Authors:** Arushi Beri, Sweta G Pisulkar, Akansha Bansod, Akshay Shrivastava, Ritul Jain, Shruti Deshmukh

**Affiliations:** 1 Department of Prosthodontics, Sharad Pawar Dental College and Hospital, Datta Meghe Institute of Higher Education and Research, Wardha, IND; 2 Department of Orthodontics and Dentofacial Orthopedics, Kalinga Institute of Dental Sciences, Bhubaneswar, IND

**Keywords:** basal bone, atrophic jaws, immediate loading, bicortical engagement, basal implants

## Abstract

In the significant atrophic jaws, it is difficult to place dental implants since there is a qualitative and quantitative shortage of future implant beds. Basal implants, also known as cortical or bicortical implants, offer a viable alternative for dental rehabilitation in patients with significant alveolar bone loss. These implants are anchored in the dense basal bone, providing immediate stability and allowing for immediate loading, thereby reducing overall treatment time and eliminating the need for extensive bone grafting procedures. This case report demonstrates the efficacy of basal implants in providing immediate functional and aesthetic restoration for patients with significant alveolar bone loss, by describing a 49-year-old patient who presented with severe alveolar ridge resorption, making traditional implant placement unfeasible. Basal implants were successfully placed, and an immediate prosthesis was delivered, resulting in excellent functional and aesthetic outcomes.

## Introduction

The ultimate goal of dental and orofacial treatments is enhancing patients' quality of life and treating oral diseases. The most frequent causes of tooth loss include trauma, periodontal disease, dental decay, tumor excision, and orthodontic therapy. These conditions have an adverse effect on patients' quality of life on an aesthetic, functional, psychological, and social level. For implant treatment, there are numerous prosthetic choices available, including All-on-4, All-on-6, Zygomatic, and Pterygoid implants. However, there ought to be enough bone in place for an implant to be placed successfully. When the requirements are not fulfilled, treatment planning for implant insertion becomes more challenging [1-5].

Since dental technology has advanced recently, implants are regarded as the best option for restoring lost teeth. Two successful implant designs and protocols that have emerged in the last few decades for the restoration of atrophic jaws are basal implants and mini dental implants. The basal implant system, which anchors the implant to the basal/cortical bone, is helpful in situations where bone grafting is not permitted because of the patient's overall health, as well as when a more cost-effective and conservative course of treatment is required. The basal cortical screw (BCS) implant is a unique basal implant; like other endo-osseous implants, it is introduced using the crestal route and is fixed deeply into the basal bone in a single piece [6].

Modern basal implantology uses implants to match the existing bone structure to quickly restore masticatory function and aesthetics. The static treatment option is determined by factors such as bone volume, density, and anatomy; the dynamic and physiological aspects of the technique are related to blood supply, muscles, occlusion, activation of stem cells with osseotensors, and functional loading of the prosthetic implant-supported teeth [7].

Two classes of basal implants, called basal osseointegrated (BOI) and BCS, are made expressly to make use of dense cortical jaw bones. Screwable basal implants (a subtype of BCS) can be placed in the socket right away following extraction thanks to their development of up to a 12 mm thread diameter [8].

Basal implants come in a variety of forms and have a distinctive design. One kind of basal implant is the crestal basal implant, which is implanted flapless and doesn't require an incision. The procedure of achieving this anchorage in basal implants is referred to as "osseo-fixation." The implants are anchored cortically. It is anticipated that secondary osseointegration will occur later, particularly in spongious bone locations where the endosseous components of the implants protrude. The primary stability, which determines the treatment outcome, is dependent upon the macro-mechanic anchorage or osseo-fixation in the second and third cortical plates [9-11].

## Case presentation

**Figure 1:**
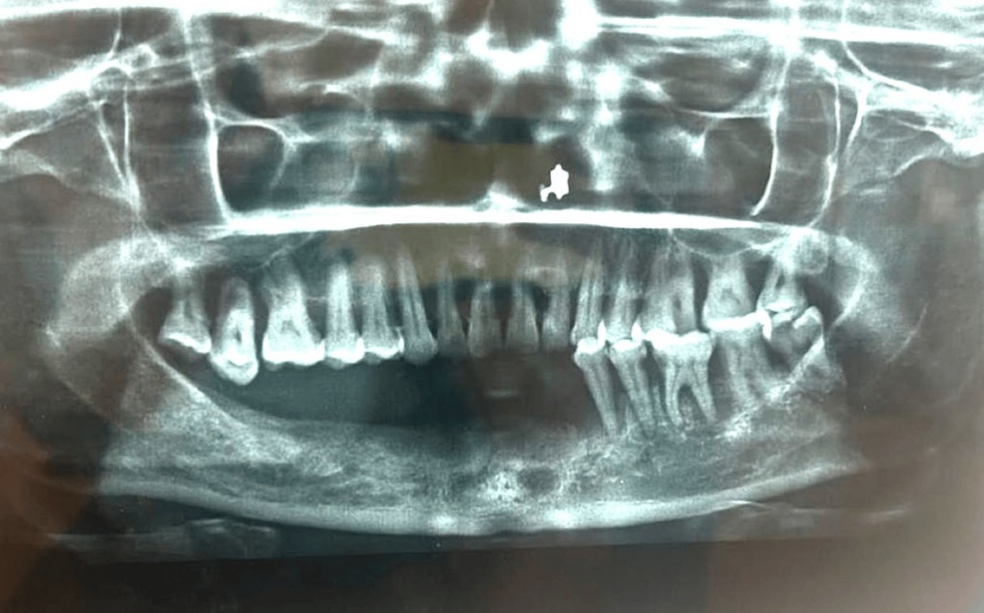
Pre-operative radiograph

A 46-year-old female patient reported to the Department of Prosthodontics with a chief complaint of difficulty of mastication and poor aesthetics due to missing teeth in the lower jaw, which led to various nutritional deficiencies and deterioration of the patient’s health over a period. A thorough clinical and radiographic evaluation was done, revealing the presence of mandibular left premolars and molars, which were periodontally compromised and had poor bone support (Figure 1).

**Figure 2:**
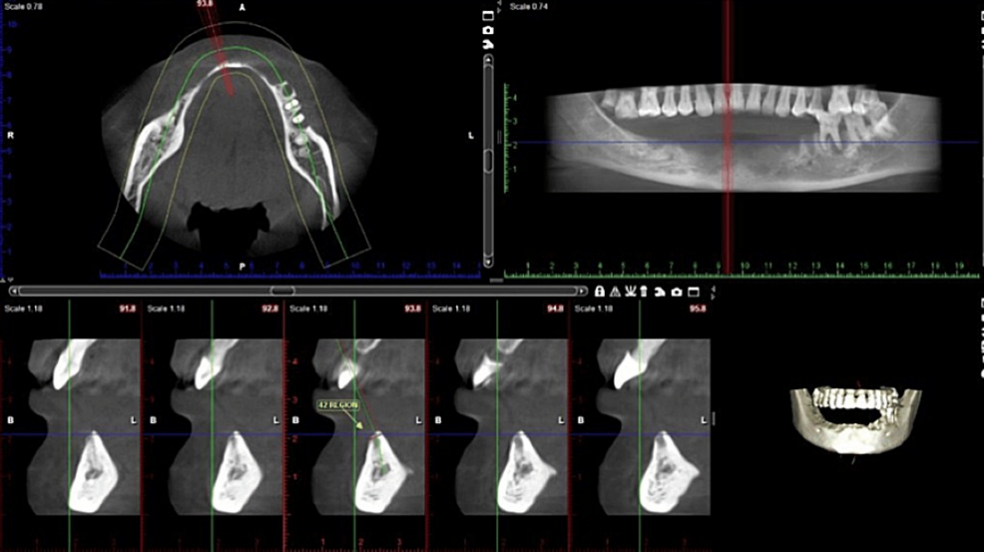
CBCT for implant planning CBCT: Cone beam computed tomography

**Figure 3:**
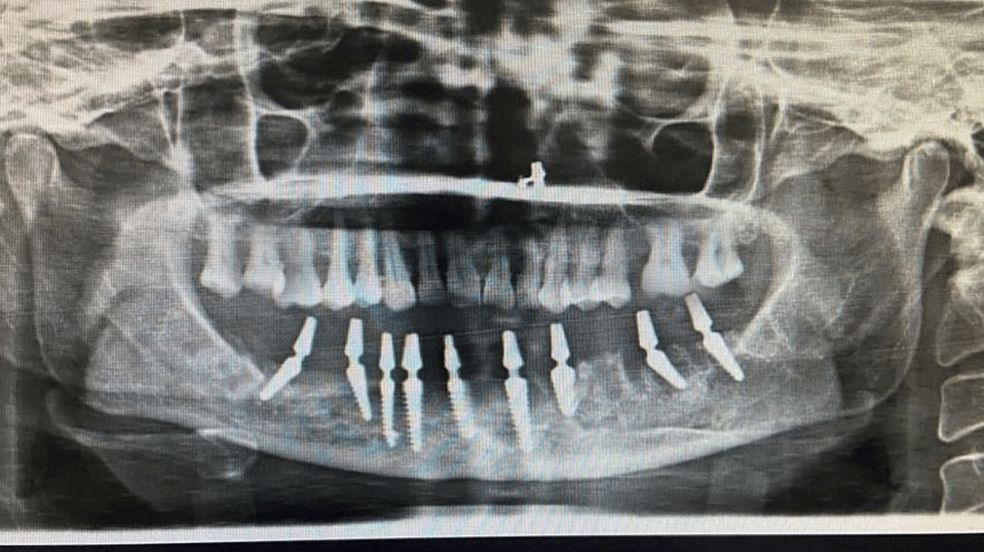
Post-operative radiograph after the placement of basal implants

**Figure 4:**
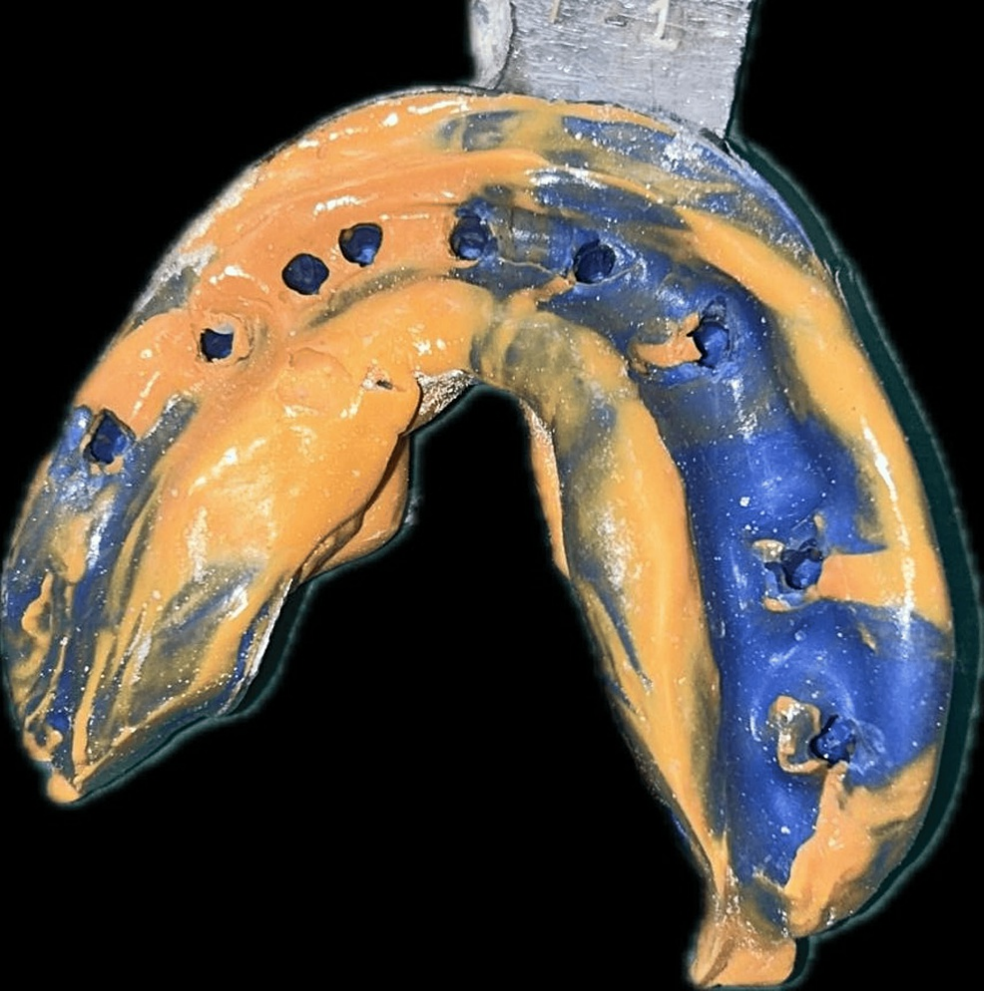
Putty light body impression of the mandibular arch

**Figure 5:**
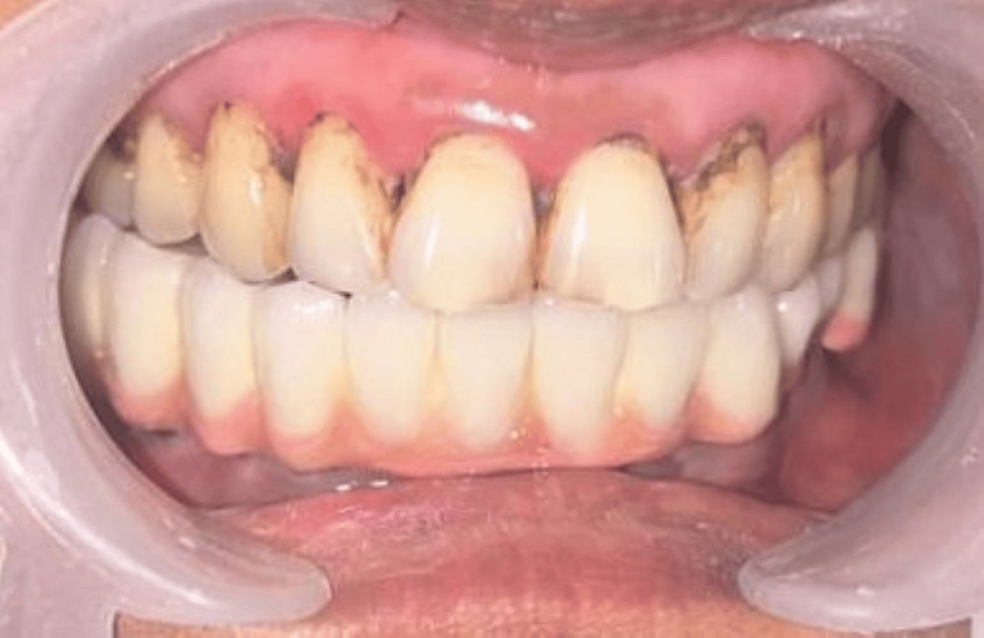
Porcelain fused to metal prosthesis after cementation

**Figure 6:**
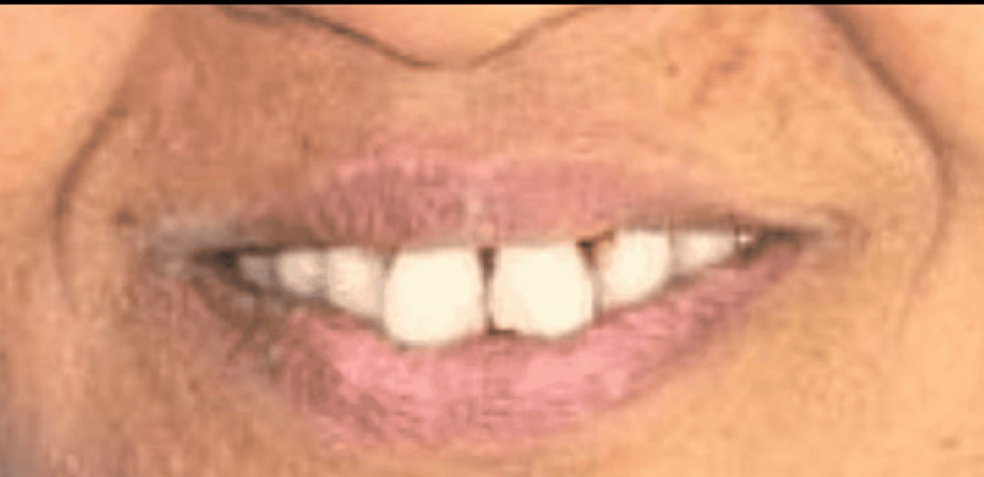
Post-operative frontal view

**Figure 7:**
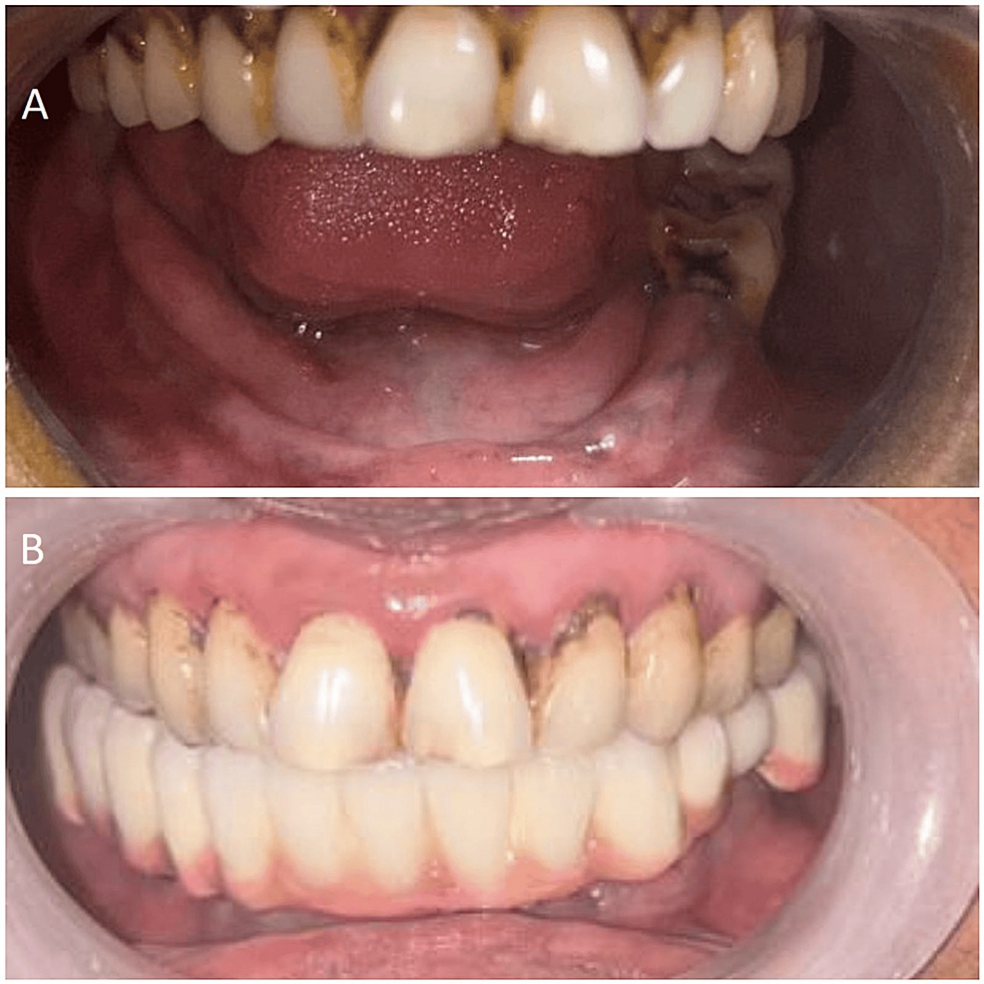
Comparison of (A) pre-operative and (B) post-operative operative images

A CBCT evaluation was done, which revealed the presence of thin bone buccolingually and mesiodistally, but the apicocoronal dimension had adequate bone (Figure 2). Additionally, the bone type was classified as D3-D4. Therefore, a treatment option involving basal implants was discussed in detail with the patient, and nine basal implants were planned for the mandibular arch (Figure 3), which were immediately loaded within 72 hours. After the placement of implants, the impression was made with putty and light body impression material (Figure 4). A jaw relation was made, and an FP3 prosthesis was planned, which was followed by metal try-in and layering with ceramic restoration and cementation of the final prosthesis (Figure 5). The implant-protected occlusal schemes were selected and any occlusal interferences in centric, protrusive, and lateral movements were removed. Figure 6 shows the post-operative frontal view of the patient. Figure 7 shows a comparison of pre-operative and post-operative operative images.

## Discussion

Dental implants have so many benefits over traditional removable and fixed tooth-supported prostheses, including the ability to replace lost teeth, that they have emerged as the most widely accepted treatment option. However, there are several drawbacks to the traditional method of placing crystal implants, the most prevalent being the design of multiunit implants, which have several interconnecting screws, delayed loading, and the need for sufficient bone volume and density. As a result, the basic dental implant design was first created by Dr. Gérard Scortecci in 1980 and underwent multiple modifications to improve into a single piece [12-15].

The alveolar bone of the jaws, which is more likely to resorb and/or disappear as teeth are extracted or as function declines, is utilized in the traditional Brånemark approach to implant insertion [16]. Conversely, basal implants are placed within the basal bone. Basal implants have several benefits because, unlike the alveolar bone, they are less likely to resorb, provide strong support for the implants, and have limited vascularity, reducing their susceptibility to infections. Biomechanically, these implants anchor in the dense basal bone, offering exceptional primary stability. Patients also benefit from fast function restoration and a shorter recovery period. The flapless technique is a less invasive treatment option that is ideal for individuals with significant bone loss, where conventional implants would fail without considerable augmentation. It also eliminates surgical damage and speeds up healing. Additionally, treatment reduces expenses and time needed, providing a more cautious approach [17-19].

Basal implants can be loaded immediately after placement because they achieve great primary stability in dense cortical bone, making them more dependable than they were previously. Restrictive splinting of the metal framework should be done as soon as feasible since the remodeling of the bone begins within 72 hours and diminishes the peri-implant bone structures. The masticatory forces generated in the bone surrounding the implants are distributed to other cortical locations by splinting [20-25].

In the present case, nine implants were inserted into the mandible. Because the mandible's bone height was insufficient, basal implant treatment was scheduled. Therefore, the use of basal implants in the case study showed that they could be very beneficial for individuals, with difficult anatomical and clinical situations. The distinct architecture of basal implants enabled a rapid loading strategy, which shortened treatment times and required fewer invasive procedures. During the follow-up period, the patient showed good functional and aesthetic outcomes, and no problems were noted. These findings highlight the potential of basal implants as a workable and practical remedy for situations involving poor bone health, providing a possible substitute for conventional implant techniques [26-28].

There are also considerations and potential challenges faced with basal implants, such as a high level of surgical expertise and precise planning. Vigilant post-operative care is essential to monitor for any signs of complications. Not all patients are candidates for basal implants; careful selection and thorough patient education are critical [29].

## Conclusions

A new and reliable therapy option for those who need to quickly restore broken ridges is basal implantology. The basal implant treatment is now accessible, safe, and affordable for all patients, including those with severe periodontitis and managed diabetes. Flapless techniques reduce bleeding, expedite recuperation times, and enhance patient outcomes even further. Based on the latest advancements in implant dentistry, which prioritize prosthetic-driven systems and rapid loading methods, these positive outcomes suggest that basal implants play a vital role in modern implantology and can greatly improve patients' quality of life. They also eliminate every disadvantage of traditional implantology.
